# Use of *Crithidia fasciculata* extract for the facile enzymatic synthesis of GDP-L-[^3^H]Fucose

**DOI:** 10.1093/glycob/cwae097

**Published:** 2024-12-21

**Authors:** Jose Carlos Paredes Franco, Maria Lucia Sampaio Güther, Michael A J Ferguson

**Affiliations:** Wellcome Centre for Anti-Infectives Research, Biological Chemistry and Drug Discovery, School of Life Sciences, Dow Street, University of Dundee, Dundee DD1 5HN, United Kingdom; Current address: Department of Biological Sciences, University of Notre Dame, Notre Dame, Indiana 46556, United States of America; Wellcome Centre for Anti-Infectives Research, Biological Chemistry and Drug Discovery, School of Life Sciences, Dow Street, University of Dundee, Dundee DD1 5HN, United Kingdom; Wellcome Centre for Anti-Infectives Research, Biological Chemistry and Drug Discovery, School of Life Sciences, Dow Street, University of Dundee, Dundee DD1 5HN, United Kingdom

**Keywords:** Crithidia fasciculata, fucose, GDP-fucose, nucleotide sugar, Trypanosomatid

## Abstract

For studies involving glycosyltransferases and nucleotide sugar transporters, radioactive nucleotide sugars are critical reagents. Of these, GDP-L-[^3^H]Fucose is currently commercially unavailable. Here, we present a facile approach for the preparation of GDP-[^3^H]-L-Fucose, using the enzymatic machinery present in the cytosol of the non-infectious and easily cultivated protozoan, *Crithidia fasciculata*, and its purification by solid phase extraction ion exchange chromatography.

## Introduction

Nucleotide sugars are essential building blocks for the synthesis of carbohydrates and glycoconjugates. Among these, guanosine 5′-diphospho-β-L-fucose (GDP-Fuc) serves as the donor substrate for the biosynthesis of fucosylated glycoconjugates, many of which are involved in biological processes including host–microbe interaction, viral infection and immunity (Thomès and Bojar 2021). In mammals, fucosylated glycans are a component of the ABO blood group and the Lewis antigen systems. These are also present in human milk oligosaccharides, which are important during development and immune protection of infants (Frohnmeyer et al 2022). In plants, fucosylated glycoconjugates play an important role in reproductive development, tissue architecture, and in their response to pathogens (Joly et al 2002; Voxeur et al 2017; Soto, Urbanowicz and Hahn 2019; Zhang et al 2019). In some intestinal bacteria, specific fucosylated epitopes have a function in molecular mimicry to evade the host immune system and maintain long-term infections (Schneider et al 2017). Recent bioinformatic analyses (Thomès and Bojar 2021) have demonstrated that fucose-containing glycans are present in all kingdoms of life, and that the ratio between fucose-containing and total glycans in different bacterial species correlates with those associated with plant roots (high ratio) or those responsible for human and animal diseases (low ratio).

Biotransformation of 6-azido-L-fucose (FucAz) to GDP-FucAz and incorporation into cellular glycoconjugates, followed by click chemistry for detection, has opened up many studies (Rabuka et al 2006; Dehnert et al 2011; Rosenlöcher et al 2015; Chen and Varki 2023). Nevertheless, GDP-[^3^H]Fuc remains as a critical tool for quantitative assays of fucosyltransferases and GDP-Fuc nucleotide sugar transporters. However, it is currently commercially unavailable.

The synthesis of nucleotide sugars in trypanosomatids, and other eukaryotes, occurs through the de novo and/or salvage pathways, depending on which enzymes are encoded in the genome and expressed in the cell (Turnock and Ferguson 2007). For the generation of GDP-Fuc, the trypanosomatids *Crithidia fasciculata* and *Leishmania major* use only a salvage pathway, since they lack the two enzymes involved in the de novo synthesis of GDP-Fuc from GDP-Man. Instead, they contain the *FKP40* and *AFKP80* genes, first identified in *L. major*, which encode bifunctional (sugar 1-kinase and pyrophosphorylase) enzymes which salvage fucose alone (fucokinase/pyrophosphorylase, FKP40) or both L-Fuc and D-arabinopyranose (D-Ara) (arabino/fucokinase/pyrophosphorylase, AFKP80) and convert them into GDP-Fuc and GDP-D-Arabinopyranose (GDP-Ara) (Guo et al 2017). The ability of AFKP80 to utilize both L-Fuc and D-Ara in the pyranose configuration most likely arises from the structural similarity of these two sugars, differing only in that L-Fuc has an extra methyl group attached to the C5 position of D-Ara*p*. These bifunctional sugar-kinase/pyrophosphorylase enzymes form cytosolic homodimers of high-molecular weight (>100 kDa) (Novozhilova and Bovin 2009). *L. major* and *C. fasciculata* use GDP-Ara for the synthesis of their major surface glycoconjugates, lipophosphoglycan (McConville et al 1990) and lipoarabinogalactan (Schneider et al 1996), respectively. Even though L-Fuc has not been directly identified in any glycoconjugate from these parasites under nomal growth conditions, when *L. major* epimastigotes or *C. fasciculata* choanomastigotes are cultured in the presence of relatively high concentrations of L-Fuc (50 mM), this sugar can substitute for D-Ara in their respective major surface glycoconjugates (Wilson et al 1999; Paredes 2023). The specific carrier(s) for L-Fuc and D-Ara in *L. major* and *C. fasciculata* have not been established, although (Ng et al 2023) recently proposed GLUT1 as a highly efficient L-Fuc transporter in mammalian cells.

A route to D-Ara from D-glucose was proposed for *C. fasciculata* involving the loss of the D-glucose C1 carbon atom (Schneider et al 1994; Schneider et al 1995; Turnock and Ferguson 2007). This route has been recently confirmed, involving both the the oxidative and non-oxidative branches of of the pentose phosphate pathway, and the conversion of D-ribulose-5-phosphate to D-arabinose-5-phophate via the isomerase activity of glutamine fructose-5-phosphate amidotransferase (Iljazi et al 2024).

Initial reports on the enzymatic synthesis of GDP-Fuc from L-Fuc involved the extraction and purification of L-Fuc kinase and GDP-Fuc pyrophosphorylase activities from extracts of pig liver, and their sequential use for the synthesis of β-L-fucose-1-phosphate (L-Fuc1-P) (7.5% yield), and then of GDP-Fuc (36.5% yield), overall yield 2.7% (Ishihara et al 1968; Ishihara and Heath 1968). These sequential syntheses were optimized by improving the enzyme isolation methodology, and adapted for the synthesis of GDP-[^3^H]Fuc from L-[^3^H]Fuc (Jabbal and Schachter 1971; Schachter et al 1972). The yields of [^3^H]L-Fuc1-P and GDP-[^3^H]Fuc were 40% and 60%, respectively, with an overall yield of 24%. Later, (Prohaska and Schenkel-Brunner 1975) reported the extraction of both activities in a crude extract of hog submaxillary glands and the conversion of L-Fuc to GDP-Fuc in a single pot reaction with an overall yield of 81% at relatively large-scale (0.1 mmoL in 100 mL). These authors included KF as a general phosphatase inhibitor to spare the L-Fuc-1P intermediate and noted substrate inhibition of the L-Fuc 1-kinase activity at >1 mM L-Fuc. Later, (Stiller and Thiem 1992) revisited the hog submaxillary gland extract reactions and added an ATP regeneration system to avoid ADP product inhibition of the L-Fuc-1-kinase reaction, and achieved an 80% yield of L-Fuc-1P. They favoured a two-step synthesis, with isolation of L-Fuc-1P prior to the pyrophosphorylase reaction, and introduced pyrophosphatase to prevent inhibition of the pyrophosphorylase step by the pyrophosphate product. The latter principle was first described by Kornberg (Kornberg 1948, 1957). Overall they were able to achieve a yield of GDP-Fuc of about 20% at the 0.05 mmoL scale. In addition to the aforementioned studies, conversion of GPD-Man to GDP-Fuc by bacterial extracts (eg. of *Agrobacterium radiobacter* or *Aerobacter aerogenes*) with about 25% yield has been demonstrated (Yamamoto et al 1984) and this could be investigated for the conversion of commercially available GDP-[^3^H]Man to GDP-[^3^H]Fuc.

Subsequent research described the synthesis of the tritium-labelled nucleotide sugar GDP-[^3^H]Ara using a kinase reaction involving a crude enzyme preparation from *C. fasciculata* lysates ([^3^H]D-Ara conversion to [^3^H]D-Ara1-phosphate ([^3^H]D-Ara1-P)) followed by chemical condensation with GMP-morpholidate ([^3^H]D-Ara1-P conversion to GDP-[^3^H]Ara) (Schneider et al 1995). Later, (Mengeling and Turco 1999) were able to perform the Ara1-kinase and GDP-Ara pyrophosphorylase reactions simultaneously by adding GTP and pyrophosphorylase to the kinase reaction mixture. Importantly, it was in this work where, based on the structural similarity between D-Ara in the pyranose configuration and L-Fuc, GDP-[^3^H]Fuc was also synthesized from L-[^3^H]Fuc using the same workflow.

Here we describe a simplified procedure, based on that of (Mengeling and Turco 1999), to synthesize and purify GDP-[^3^H]Fuc and to recycle unreacted [^3^H]Fuc. This method utilizes a crude enzymatic preparation from *C. fasciculata* lysates that remains active for at least one week, combined with solid-phase extraction (SPE) ion exchange chromatography.

## Results

In order to investigate processes such as the recently discovered mitochondrial fucosylation in the trypanosomatid parasites (Bandini et al 2021; Guo et al 2021; Paredes Franco et al 2023), access to GDP-[^3^H]Fuc is critical. However, during the characterization of the *Trypanosoma cruzi* mitochondrial fucosyltransferase (Paredes Franco et al 2023), we found that GDP-[^3^H]Fuc was no longer commercially available to use as the donor substrate in radioactive fucosyltransferase assays. Therefore, we used the procedure of (Mengeling and Turco 1999) to make GDP-[^3^H]Fuc from [^3^H]Fuc using a crude enzyme preparation from *C. fasciculata*. In doing so, we made some modifications that we think make the procedure slightly simpler and which may be of value to others needing to make and purify GDP-[^3^H]Fuc.

**Fig. 1:**
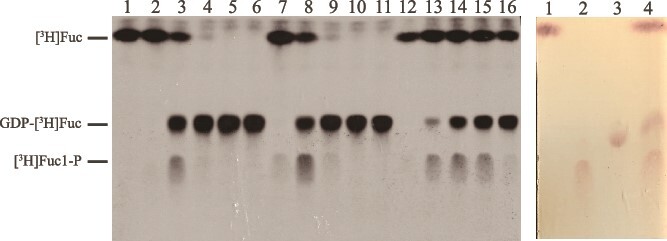
Small scale GDP-[^3^H]Fuc syntheses. Small-scale (1 μCi [^3^H]Fuc) syntheses of GDP-[^3^H]Fuc were followed over time (t = 0, 5, 15, 30, 60 min) and analyzed by HPTLC and fluorography (left panel) using fresh *C. Fasciculata* crude enzyme preparation (lanes 2–6), or *C. Fasciculata* crude enzyme preparation stored at 4 °C for 7 days (lanes 7–11) or stored frozen at -20 °C with 10% glycerol for 7 days and thawed (lanes 12–16). An authentic standard of [^3^H]Fuc was also run (lane 1). The HPTLC chromatographic positions of authentic standards of unlabeled Fuc (lane 1), Fuc1-P (lane 2), GDP-Fuc (lane 3) and a mixture of all three (lane 4) were determined by orcinol/H_2_SO_4_ staining (right panel).

In small (1 μCi [^3^H]Fuc) scale experiments we observed almost 100% conversion of [^3^H]Fuc to GDP-[^3^H]Fuc within 30 min using a fresh *C. fasciculata* crude enzyme preparation that had been treated with protease inhibitors, fractionated by size using a simple 100 kDa cut-off centrifugal membrane filtration step, exchanged into a buffer containing NaF to reduce phosphatase degradation of the [^3^H]Fuc1-P intermediate and supplemented with yeast pyrophosphatase **(**Fig. 1**, left panel, lanes 2–6)**. We further assessed the stability of this *C. fasciculata* crude fucokinase/GDP-Fuc pyrophosphorylase preparation and found that it was stable for a week when kept at 4 °C **(**Fig. 1**, left panel, lanes 7–11)** and reasonably (but less) stable after being frozen with 10% glycerol at −20 °C and thawed a week later **(**Fig. 1**, left panel, lanes 12–16).**

**Fig. 2:**
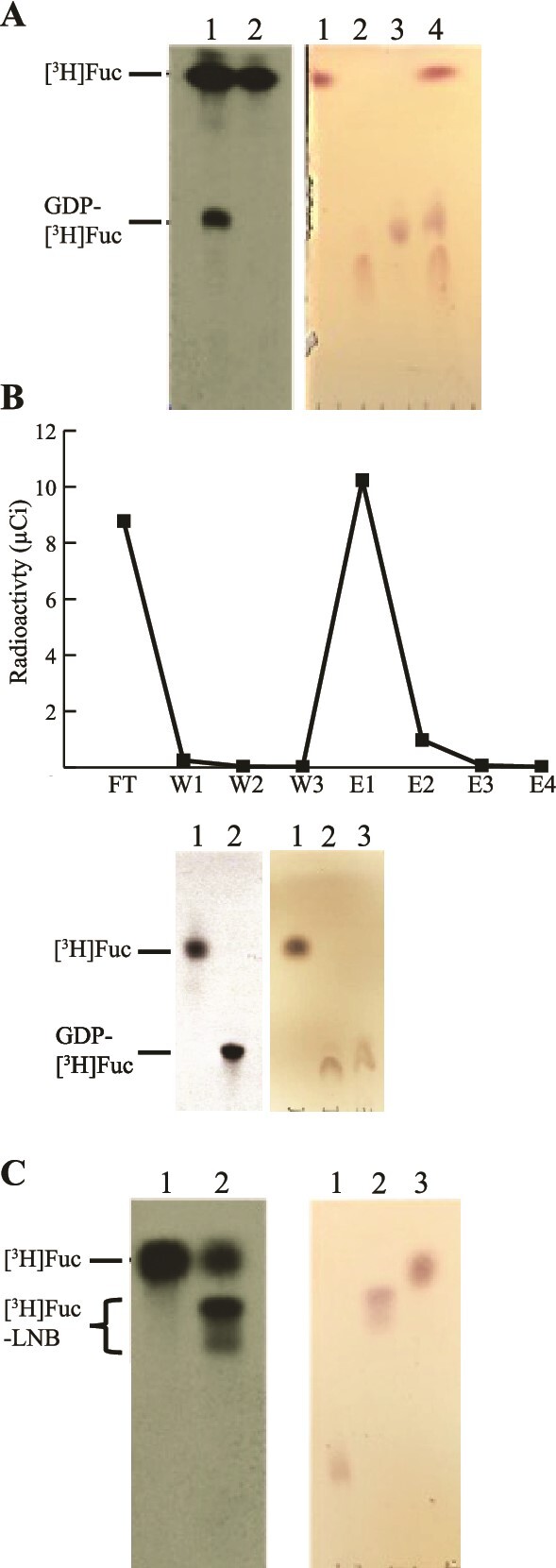
Large scale synthesis, purification and use of GDP-[^3^H]Fuc. (A) Large scale (50 μCi [^3^H]Fuc) synthesis of GDP-[^3^H]Fuc using fresh *C. Fasciculata* crude enzyme preparation. Comparison of the fluorographic signals (left panel) from the GDP-[^3^H]Fuc synthesis reaction (lane 1) and 50 nCi of [^3^H]Fuc standard (lane 2) indicate that partial conversion of [^3^H]Fuc to GDP-[^3^H]Fuc was achieved. Authentic standards of unlabeled Fuc (lane 1), Fuc1-P (lane 2), GDP-Fuc (lane 3) and a mixture of all three (lane 4) were run in parallel and their position determined by orcinol/H_2_SO_4_ staining (right panel). (B) Analysis of GDP-[^3^H]Fuc synthesis using [^3^H]Fuc recovered from previous reactions (see panel A). WAX-SPE purification profile (top) and HPTLC analysis by fluorography (bottom, left panel) of fractions FT (lane 1) and E1 (lane 2) after purification. A parallel run of orcinol/H_2_SO_4_-stained authentic standards (bottom, right panel) of Fuc (lane 1), Fuc1-P (lane 2), and GDP-Fuc (lane 3) confirmed the identities of the purified radioactive compounds. (C) *E. Coli*-expressed rTbFUT1 activity assay using purified GDP-[^3^H]Fuc from (B). Radioactive products detected by HPTLC and fluorography (left panel) indicate the successful generation of radiolabeled product ([^3^H]Fucα1-2Galβ1-3GlcNAc) in the presence (lane 2) but not the absence (lane 1) of the LNB acceptor disaccharide. In contrast, only free [^3^H]Fuc could be detected when the LNB acceptor disaccharide was not included (lane 1). The chromatographic positions of unlabeled standards (right panel) were determined by HPTLC and orcinol/H_2_SO_4_ staining; GDP-Fuc (lane 1), LNB (lane 2) and Fuc (lane 3).

In larger (50 μCi [^3^H]Fuc) scale reactions, we increased the reaction volume and amount of *C. fasciculata* crude enzyme preparation supplemented with pyrophosphorylase 4-fold and increased the incubation time to 90 min. Analysis of the reaction mixtures indicated incomplete conversion into GDP-[^3^H]Fuc in this case **(**Fig. 2A**)** and, following WAX-SPE separation of unreacted [^3^H]Fuc from GDP-[^3^H]Fuc product, we were able to measure the yield of GDP-[^3^H]Fuc as 18.6%. A sample (50 μCi) of the free [^3^H]Fuc recovered from the WAX-SPE separations was recycled into a further reaction for 180 min followed by WAX-SPE separation and this time the yield of GDP-[^3^H]Fuc was 56% **(**Fig. 2B).

Because these 50 μCi scale reactions furnished enough GDP-[^3^H]Fuc for our needs, we did not further optimize this scale of reaction. However, we would recommend that future syntheses either further extend the incubation time, or return to the GDP-[^3^H]Fuc: *C. fasciculata* crude enzyme and reaction buffer ratios of the small scale experiments, and/or consider using more ATP or an ATP regeneration system in the reaction buffer. The latter recommendation is based on the absence of [^3^H]Fuc1-P in the reaction analysis **(**Fig. 2A**),** which suggests that the conversion of [^3^H]Fuc to [^3^H]Fuc1-P may be the rate limiting step.

Finally, in order to demonstrate that our “home-made” GDP-[^3^H]Fuc was useable, we used it as the sugar donor in a fucosyltransferase assay employing *E. coli*-expressed rTbFUT1 (Bandini et al 2021). The results reproduced the reported activity of rTbFUT1 (Bandini et al 2021). Thus, in the absence of an LNB disaccharide acceptor substrate, rTbFUT1 transfers [^3^H]Fuc from GDP-[^3^H]Fuc to water, producing free [^3^H]Fuc **(**Fig. 2C**, left panel lane 1).** However, in the presence of a preferred substrate (i.e. the LNB disaccharide acceptor), the production of free [^3^H]Fuc is reduced and, instead, rTbFUT1 transfers [^3^H]Fuc from GDP-[^3^H]Fuc to LNB to form the Fucα1-2Galβ1-3GlcAc trisaccharide product which migrates on HPTLC just below LNB itself **(**Fig. 2C**, left panel lane 2).** Two bands are observed because the α- and β-anomers of LNB and [^3^H]Fuc-LNB separate on this TLC system. The GDP-[^3^H]Fuc synthesized following the procedure described in this paper has also been successfully tested in fucosyltransferase assays of endogenously overexpressed and immunoprecipitated *T. cruzi* mitochondrial fucosyltransferase (TcFUT1) (Paredes Franco et al 2023).

## Discussion

The synthesis of nucleotide sugars typically involves advanced chemical methods or the expression and purification of multiple enzymes from the GDP-Fuc salvage and de novo pathways, and others to aid in the process, (Wang et al 2019; Mahour et al 2021; Frohnmeyer et al 2022). The use of enzyme cascades for the synthesis of GDP-Fuc and other nucleotide sugars have been recently reviewed (Frohnmeyer and Elling 2023). These approaches can be challenging for researchers who only require access to tiny amounts of material in radiolabelled form (Mikkola 2020; Rexer et al 2021; Dolan et al 2022). Here, we have further simplified a facile approach for producing radiolabeled GDP-[^3^H]Fuc (Mengeling and Turco 1999) which harnesses enzyme activities found in the cytosol of *C. fasciculata*, a protozoan insect parasite that poses no health threat to humans, and that is easily cultured without the need for serum supplementation or carbon dioxide incubation.

## Materials and methods

### Cell culture

*C. fasciculata* HS6 strain (Shim and Fairlamb 1988) were grown in SDM-79 medium (Brun and Schönenberger. 1979) supplemented with 2 g/L NaHCO_3_, 1 X GlutaMAX as L-glutamine source, and 20 μg/mL hemin (added from an 10 mg/mL solution in in 50 mM NaOH) instead of 7.5 μg/mL as in the original formula. Culture flasks were non-tissue culture treated and non-vented and cells were grown at 28 °C, with no need of CO_2_. Parasite growth was monitored by hemocytometer and cells were grown for 4–5 days up to late log phase (1 × 10^7^ cells/mL) before being harvested or diluted to 1 × 10^4^ cells/mL for subculture. Cell division time was typically 9–10 h, reaching stationary phase at 1.5–1.8 × 10^7^ cells/mL. For cryo-preservation of the cells, they were grown to mid-late log phase (5 × 10^6^ to 1 × 10^7^ cells/mL) and 700 μL cell culture aliquots were mixed with 100 μL 80% of sterile glycerol solution in 1 mL cryogenic vials with external thread. These were then transferred to a − 80 °C freezer for short-term storage, or liquid nitrogen tank for long-term storage.

### *C. Fasciculata* crude enzyme preparation

The following protocol was developed to make the crude enzyme preparation: *C. fasciculata* cells (1 × 10^9^ cells) were harvested in late log phase by centrifugation (1,000 × g, 10 min, 4 °C), washed twice with phosphate buffered saline (137 mM NaCl, 2.7 mM KCl, 10 mM Na_2_HPO_4_, and 1.8 mM KH_2_PO_4_, pH 7.2) and then resuspended in 600 μL 10 mM Tris–HCl pH 7.5, 1X EDTA-free protease inhibitor cocktail (Roche). Resuspended cells were lysed with two cycles of freeze-thawing by placing in dry ice for 15 min and then holding tubes under cold water stream until material was thawed again. Lysate was then centrifuged (16,000 x g, 10 min, 4 °C) and the supernatant centrifuged again under the same conditions. The supernatant was transferred to a 1 mL syringe and filtered using a 0.45 μm Millex-HV PVDF syringe filter (Merck). The filtrate was split into two equal portions and each transferred to a 100 kDa cut-off, 0.5 mL capacity, Amicon Ultra-0.5 centrifugal filter device (Merck). These were centrifuged (12,000 × g, 7.5 min, 4 °C) and the flow-through discarded. The concentrated retentates were then buffer-exchanged by the addition of the following, with centrifugation after each addition: two additions of 400 μL of ice-cold 10 mM Tris–HCl pH 7.2, 1X EDTA-free protease inhibitor cocktail, one addition of ice-cold 400 μL of ice-cold 10 mM Tris–HCl pH 7.2, and one addition of 400 μL of ice-cold 10 mM Tris–HCl pH 7.2, 20 mM NaF. In all cases, centrifugation was at 12,000 × g, 10 min, 4 °C and flow-through material was discarded. The final retentate was recovered by washing the device membranes twice with 100 μL of ice-cold 10 mM Tris–HCl pH 7.2, 20 mM NaF and combining them in a total volume of 300–350 μL. The typical total protein yield was 2 mg/mL. In this way, salts, small molecule metabolites and proteins <100 kDa (including proteases, phosphatases and phosphodiesterases) were removed from, and the phosphatase inhibitor sodium fluoride (NaF) (NagDas and Bhattacharyya 1984; Schneider et al 1995) (to protect the Fuc1-P intermediate) was introduced into, the crude enzyme preparation.

### Synthesis of GDP-[^3^H]Fuc

The radiolabeled starting material, tritiated fucose ([^3^H]Fuc), was purchased from American Radiolabeled Chemicals as a 1 mCi/ml solution in ethanol /water (9:1, v:v) with a specific activity of 60 Ci/mmoL.

For small-scale reactions, aliquots of 1 μCi [^3^H]Fuc were dried in PCR tubes under a gentle stream of N_2_ and redissolved in 2.5 μL of reaction buffer (20 mM MgSO_4_, 8 mM ATP, 8 mM GTP dissolved in 10 mM Tris–HCl pH 7.2). In another tube, 1 μL of 400 mU/μL of yeast inorganic pyrophosphatase (Sigma) was added to 9 μL of *C. fasciculata* crude enzyme preparation. Pyrophosphatase was added to prevent pyrophosphate product-inhibition of the pyrophosphorylase activity (Kornberg 1948, 1957; Ferjani et al 2018). Converting pyrophosphate to inorganic phosphate thus shifts the equilibrium of the pyrophosphorylase reaction towards GDP-Fuc production. Aliquots (2.5 μL) of this *C. fasciculata* crude enzyme preparation plus pyrophosphatase mix were added to the [^3^H]Fuc aliquots to give a total volume of 5 μL in each PCR tube. Reactions were performed at 30 °C and 0.8 μL samples were taken at different times to analyze the reaction. These samples were added to 1 μL aliquots of 100 mM EDTA on ice to stop the reaction. Aliquots, 1 μL of 1 μg/μL each of L-Fuc, Fuc1-P and GDP-Fuc (all from Sigma), were added to act as carriers to aid subsequent chromatography, and 2 μL of 40% 1-propanol was added to facilitate transfer to high-performance thin layer chromatography (HPTLC) plates.

For large-scale synthesis, 50 μCi of dried [^3^H]Fuc was dried under N_2_ and redissolved in 10 μL of reaction buffer. At the same time, 18 μL of fresh *C. fasciculata* crude enzyme preparation was mixed with 2 μL of 400 mU/μL yeast inorganic pyrophosphatase and half (10 μL) was added to the [^3^H]Fuc in reaction buffer. The 20 μL reaction mixture was incubated at 30 °C for 90 or 180 min after which 380 μL of ice-cold 2 mM EDTA was added to stop the reaction. Reaction products were analyzed by HPTLC, as described above, after mixing 1 μL of the reaction with 1 μL of 1 μg/μL each of L-Fuc, Fuc1-P and GDP-Fuc as non-radioactive carriers and 2 μL of 40% 1-propanol.

### High-performance thin layer chromatography (HPTLC)

For the analysis of non-radioactive and [^3^H]-labelled sugars and sugar nucleotides, chromatography was performed on aluminum-backed silica gel 60 HPTLC plates (Merck). Samples were dissolved in (in the case of radioactive samples) or adjusted to (in the case of non-radiolabeled samples) 20% to 40% 1-propanol and each sample was applied to the HPTLC plate origin 1–2 μL at a time, allowing each application to dry before adding the next. The HPTLC plates were developed twice with 1-propanol, acetone, water (9:6:4, v:v:v) as mobile phase. All solvents used were AnalaR NORMAPUR grade (VWR). Plates were either stained with orcinol/H_2_SO_4_ reagent or subjected to fluorography to detect non-radioactive and radioactive sugars, sugar phosphates and nucleotide sugars.

### Orcinol/H_2_SO_4_ staining

To stain non-radiolabeled carbohydrates, HPTLC plates were sprayed using an Aldrich flask-type sprayer (Merck) with an orcinol/H_2_SO_4_ solution, allowed to dry for 10 min, and then heated with a heat gun (Steinel HL1610 S) until the spots appeared. The staining solution was prepared by dissolving 180 mg of orcinol (Merck) in 5 mL of water, and then adding 75 mL absolute ethanol. After placing solution on ice, 10 mL concentrated H_2_SO_4_ (18.4 M) was added dropwise and carefully mixed in. The final solution can be stored at 4 °C, protected from light, and brought to room temperature before use.

### Fluorography of TLC plates

Due to the weak β particles emitted by the [^3^H] isotope, indirect autoradiography, or fluorography, was necessary to visualize the different [^3^H]-labelled sugars and sugar nucleotides present in the HPTLC plates. After chromatography, silica HPTLC plates were sprayed three times with EN^3^HANCE Liquid Autoradiography Enhancer (PerkinElmer), leaving plates to dry between each spraying for 15 min. After the final spraying the plates were allowed to dry completely and were placed inside an exposure cassette against an X-ray film (Sigma Carestream Biomax XAR film) and a BioMax TranScreen LE intensifying screen (Kodak). Exposure cassettes were left at −80 °C from a few days to several weeks, depending on the amounts of radioactivity being detected. The films were developed on a Protec ECOMAX X Ray Film Processor in a dark room.

### Ion exchange chromatography for the recovery of GDP-[ ^3^H]Fuc and unreacted [ ^3^H]Fuc

A 1 mL weak anion exchange solid phase extraction (WAX-SPE) cartridge with 30 mg sorbent (Waters Oasis) was used. The WAX-SPE sorbent materal carries a positive charge and is designed to bind negatively charged compounds, such as in our case: GMP, GTP, ADP, ATP, Fuc-1-P and GDP-[^3^H]Fuc, while allowing neutral compounds, such as free [^3^H]Fuc, pass straight through. The bound negatively charged compounds can then be eluted with salt, in our case 1 M ammonium acetate which can be conveniently removed by freeze drying. The WAX-SPE cartridges were prepared by passing through it 0.5 mL water, followed by 0.5 mL of 1 M ammonium acetate (four times) and 0.5 mL water. Large-scale GDP-[^3^H]Fuc synthesis reactions were passed through the cartridge and the eluate recovered, along with two washes of 150 μL water. This was the flow-through (FT) fraction. Next the cartridge was washed three times with 0.5 mL water and these wash eluates (W1–W3) were collected separately. Finally, four eluates (E1–E4) with 0.5 mL 1 M ammonium acetate were collected. Aliquots of the eight fractions were taken for liquid scintillation counting. Unreacted [^3^H]Fuc appeared in the FT fraction since, being an uncharged molecule, it did not interact with the positively-charged stationary phase. Synthesized GDP-[^3^H]Fuc appeared mostly in fraction E1, with a small portion appearing in fraction E2 in a few experiments.

Fraction E1 was frozen by placing it on dry ice, and then freeze-dried. Dried material was redissolved in 100 μL water and freeze-dried again. This process removed the majority of the volatile ammonium acetate, as judgesd by the lack of visible residue. The desalted GDP-[^3^H]Fuc was dissolved in 50% ethanol and stored at −20 °C. The removal of ammonium acetate by freeze-drying allowed analysis by HPTLC.

Fraction FT was desalted by passage through a mixed-bed column made of 100 μL each of the ion exchange resins Chelex 100 (Na^+^), Dowex AG50 X12 (H^+^), Dowex AG4X4 (OH^−^) and QAE-Sephadex A25 (OH^−^), layered in that order from top to bottom. The column was pre-washed with 10 column volumes (4 mL) of water. Samples were loaded and then eluted three times with 0.4 mL water each time. Eluates were pooled together. The total eluate was then aliquoted into microcentrifuge tubes and dried in a centrifugal vacuum dryer at 50 °C for 3–4 h. Dried material was recovered and combined by washing the tubes twice with 70% ethanol. This stock of desalted [^3^H]Fuc for re-use was stored at −20 °C.

### Recombinant *Trypanosoma brucei* mitochondrial fucosyltransferase (rTbFUT1) activity assay

Procedures described in (Bandini et al 2021) were followed for the expression and purification of recombinant N-terminally GST-tagged TbFUT1 (rTbFUT1) using pGEX6P1-GST-PP-TbFUT1 transformed *Escherichia coli*. The fucosyltransferase assay and HPTLC analysis of products was also according to (Bandini et al 2021), using 2 μg of affinity-purified GST-TbFUT1 per assay, except that 0.4 μCi of our custom made GDP-[^3^H]Fuc was used per assay and the final concentration of lacto-N-biose (LNB) acceptor substrate was 1 mM. Briefly, the assay involves the TbFUT1 catalysed transfer of [^3^H]Fuc from the GDP-[^3^H]Fuc nucleotide sugar donor to a the LNB (Galβ1-3GlcNAc) acceptor disaccharide, to form the [^3^H]Fucα1-2Galβ1-3GlcNAc trisaccharide product that is resolved by HPTLC and detected by fluorography.

## Data Availability

The data pertaining to this study that is not included here will be made available upon request to the corresponding author.
